# Does Lin28 Antagonize miRNA-Mediated Repression by Displacing miRISC from Target mRNAs?

**DOI:** 10.3389/fgene.2012.00240

**Published:** 2012-11-16

**Authors:** Amanda N. Kallen, Jing Ma, Yingqun Huang

**Affiliations:** ^1^Department of Obstetrics, Gynecology and Reproductive Sciences, Yale Stem Cell Center, Yale University School of MedicineNew Haven, CT, USA; ^2^Obstetrics and Gynecology Department, Third Xiangya Hospital of Central South UniversityChangsha, Hunan, China

**Keywords:** Lin28, miRNA, translation, oncogene, stem cell, synaptic

## Abstract

Lin28 is a developmentally regulated RNA-binding protein that plays important roles in diverse physiological and pathological processes including oncogenesis and brain synaptic function. These pleiotropic roles of Lin28 are mechanistically linked both to its ability to directly stimulate translation of genes involved primarily in cell growth and metabolism and to its ability to block biogenesis of a subset of miRNAs including the let-7 family of miRNAs. In the case of direct stimulation of gene expression, Lin28 binds to targeted mRNAs through recognition of Lin28-responsive elements (LREs) within mRNAs and recruits RNA helicase A (RHA) to promote translation. RHA belongs to the DEAD-box protein family of RNA helicases, which generally catalyze ATP-dependent unwinding of RNA duplexes or remodeling of ribonucleoprotein complexes (RNPs). Since any given mRNA can potentially be inhibited by miRNAs bearing complementary sequences, we hypothesize that binding of Lin28 to LREs not only nucleates the binding of multiple Lin28 molecules to the same mRNA, but also leads to remodeling of RNPs through recruitment of RHA and causes release of inhibitory miRNA-induced silencing complexes bound to the mRNA. This mode of action may contribute to Lin28-mediated stimulation of translation in both tumor and neuronal cells.

## Lin28 in Cancer

Lin28 is an evolutionarily conserved RNA-binding protein that has been implicated in diverse processes ranging from development, pluripotency, metabolism and cancer, to brain synaptic function (reviewed in Huang, 2012; Thornton and Gregory, 2012). The expression of Lin28 is developmentally regulated: it is expressed widely in early stage embryos, with expression diminishing and becoming limited to a few tissues after birth (Yang and Moss, 2003). Despite its high expression in human embryonic stem (ES) cells, and despite the fact that it is essential for ES cell viability and pluripotency, Lin28 is not expressed in most adult tissue cells (reviewed in Huang, 2012). However, aberrant activation of Lin28 (and its paralog Lin28B) is found in a variety of human malignancies and is associated with advanced disease states and poor prognosis (Lu et al., 2009; Viswanathan et al., 2009; King et al., 2011; Feng et al., 2012, and reviewed in Huang, 2012; Thornton and Gregory, 2012).

It is well established that Lin28 promotes malignancy through both indirect and direct mechanisms. The former involves let-7 miRNA-mediated repression of oncogenes and cell growth-promoting genes that contain complementary sequences to let-7 (hence let-7 target genes), which is counteracted by Lin28 via blockage of let-7 production (reviewed in Huang, 2012; Thornton and Gregory, 2012). The latter involves direct binding of Lin28 to its own target genes, thereby promoting their translation (Polesskaya et al., 2007; Xu and Huang, 2009; Xu et al., 2009; Qiu et al., 2010; Jin et al., 2011; Peng et al., 2011; Feng et al., 2012). Recent immunoprecipitation and genome-wide deep sequencing studies have shown that in human ES cells ∼5% of polyadenylated mRNAs are bound and regulated by Lin28, including those encoding metabolic enzymes and ribosomal proteins whose expression levels are known to be coupled to cell growth and metabolism (Peng et al., 2011). Importantly, this set of genes overlaps significantly with genes related to glucose, insulin, and diabetes which have been identified by Gene Set Enrichment Analysis as potential direct targets of Lin28 regulation (Zhu et al., 2011). Thus, this direct mechanism is believed to contribute critically to Lin28’s ability to affect pluripotency, cell growth, and metabolism (reviewed in Huang, 2012). This direct mechanism, coupled with the indirect mechanism mediated by let-7, is essential for maintaining the undifferentiated, highly proliferative and metabolic states, a characteristic of many tumor cells.

## Lin28 in Brain Synaptic Function

Target-specific regulation of translation in response to stimuli plays a key role in neuronal function and synaptic plasticity. Lin28 was recently reported to contribute, in an indirect fashion, to target-specific induction of protein synthesis by brain-derived neurotrophic factor (BDNF). Specifically, in terminally differentiated neurons, BDNF elevates Lin28 protein levels rapidly, and independently of transcription. This leads to selective loss of Lin28-regulated miRNAs including let-7, thus leading to increased translation of a specific set of genes targeted by these miRNAs (Huang et al., 2012). Based on the fact that Lin28 directly stimulates translation of its own target mRNAs in multiple cell types (Li et al., 2012, and reviewed in Huang, 2012), it is conceivable that Lin28 may also stimulate translation in response to BDNF in neuronal cells, which is an intriguing possibility that warrants further investigation.

## Lin28-Mediated Stimulation of Translation

How does Lin28 stimulate translation? While the mechanisms by which Lin28 blocks the biogenesis of let-7 and those underlying let-7-mediated inhibition of translation have been extensively studied (reviewed in Djuranovic et al., 2011; Fabian and Sonenberg, 2012; Thornton and Gregory, 2012), the mechanism(s) by which Lin28 directly stimulates translation of target mRNAs are not well understood.

### mRNA target recognition by Lin28

**Figure 1:**
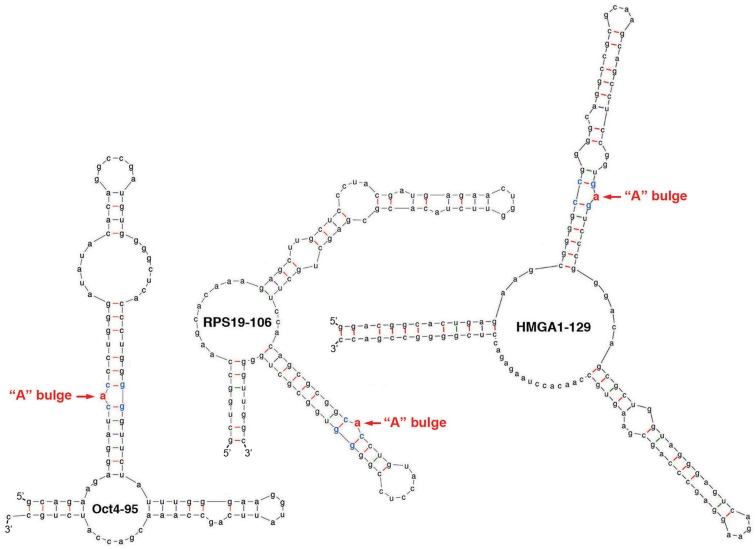
**Structural characteristics of LREs**. Shown are computationally predicted secondary structures of LREs derived from ORFs of three Lin28 targets Oct4, RPS19, and HMGA1. The critical “A” bulges are highlighted in red.

It has been estimated that approximately 5% of cellular mRNAs are bound and regulated by Lin28 at the post-transcriptional level (reviewed in Huang, 2012). How does Lin28 select its target mRNAs? What are the features in the mRNAs that confer specificity for recognition? Lin28-binding sites, or Lin28-responsive elements (LREs), have been found in various regions of transcripts including open reading frames (ORFs) and 5′- and 3′-UTRs, with the LREs found in ORFs being relatively better characterized (reviewed in Huang, 2012). These LREs occur in a range of sequences and sizes ranging from 95-nt to 456-nt long. Systematic and stepwise sequence narrowing down approaches, as well as more specific analysis of sequence alignments between target mRNAs, between LREs, and across species, have so far failed to identify any recognition consensus sequences. However, computational RNA structure prediction studies combined with *in vitro* binding and *in vivo* reporter gene analysis identified one unique sequence and structural motif that is shared by multiple ORF-localized LREs (Lei et al., 2011). This motif is characterized by an “A” bulge flanked by two G:C base-pairs embedded in a complex secondary structure (Figure 1). Remarkably, in every case tested, a single nucleotide substitution or deletion of this “A” residue results in loss of Lin28-binding and translational stimulation (Lei et al., 2011). It remains to be determined whether this motif is common to most or all LREs and how the detailed and higher-order structures of this motif in complex with Lin28 would look like.

### RHA as a co-factor of Lin28

**Figure 2:**
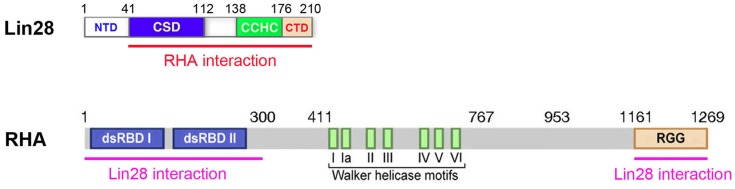
**Schematic diagram of Lin28 and RHA interaction domains**. Numbers are in amino acids. NTD, N-terminus domain; CSD, cold-shock domain; CCHC, retroviral-type CCHC (cys-cys-his-cys) zinc finger-containing domain; CTD, C-terminus domain; dsRBD, double-stranded RNA-binding domain; Walker helicase motifs, motifs of conserved DEAD-box RNA helicases; RGG, domain rich in arginine-glycine-glycine repeats. Both the N- and C-terminus domains (underlined in pink) of RHA interact with Lin28. The 41-aa NTD of Lin28 is dispensable for these interactions. However, a mutant Lin28 missing the 35-aa CTD not only fails to interact with RHA, but also exerts a dominant-negative effect on Lin28-dependent stimulation of translation.

A connection between Lin28 and RNA helicase A (RHA) was first uncovered in a co-immunoprecipitation and mass spectrometry study using human ES cells, where RHA was found to be significantly enriched in Lin28-containing protein complexes (Qiu et al., 2010). This Lin28-RHA interaction was insensitive to RNase treatment, suggesting a direct interaction that was not bridged by RNA, despite the fact that both proteins are RNA-binding proteins. It was later found that the interaction also occurs in other cell types (Jin et al., 2011). Further studies have mapped the interaction domains of both proteins (Jin et al., 2011). *In vitro* GST pull-down experiments using bacterially expressed RHA fragments fused to GST and Flag-tagged Lin28 expressed from HEK293 cells demonstrated that the C-terminal domain (CTD) of Lin28 is required for interaction with RHA at both its N- and C-terminal regions (Figure 2). These interactions were further confirmed by co-IP studies using Flag-tagged Lin28 and RHA domains expressed in HEK293 cells. As all studies were performed using crude cell lysates, the possibility that the Lin28-RHA interaction might be bridged by other factor(s) cannot be excluded (Jin et al., 2011).

What is the biological significance of this Lin28-RHA interaction? Does it contribute to Lin28-dependent stimulation of translation? Indeed, when RHA was down-regulated by siRNAs, Lin28-dependent stimulation of LRE-containing mRNAs was impeded (Qiu et al., 2010). Also, a mutant Lin28 missing the CTD (see Figure 2) was able to bind RNA but failed to interact with RHA or to stimulate translation. Moreover, this mutant inhibited Lin28-dependent stimulation of translation of LRE-containing mRNAs when co-expressed with wild-type Lin28, hence a dominant-negative effect (Jin et al., 2011). Further, there existed a positive correlation between Lin28 protein levels and the extent of RHA association with polysomes, suggesting that Lin28 actively recruits RHA to the translational machinery to facilitate target mRNA translation (Jin et al., 2011). Taken together, these observations strongly support a role of RHA in Lin28-mediated stimulation of translation. Then, how does Lin28-RHA interaction promote translation?

### RHA-dependent stimulation of translation

RHA is a member of the conserved DEAD-box protein (DBP) family of RNA helicases that function in diverse aspects of RNA metabolism including transcription, splicing, nuclear export, and translation (reviewed in Jarmoskaite and Russell, 2011). By separating strands of short RNA duplexes using energy from ATP, DBPs destabilize localized structural elements within long RNA molecules and facilitate new interactions, thereby promoting rearrangements and remodeling of ribonucleoprotein complexes (RNPs; reviewed in Jarmoskaite and Russell, 2011). For instance, the eIF4A and Ded1 helicases promote ATP-dependent disruption of secondary structures within mRNAs to facilitate translation initiation (Svitkin et al., 2001; Marsden et al., 2006). Likewise, RHA is required for translation of selected mRNAs that harbor highly structured elements (post-transcriptional control elements or PCEs) in their 5′-UTRs (Hartman et al., 2006; Bolinger et al., 2010). It is believed that PCEs act as barriers to ribosomal scanning and that binding of RHA to PCEs disrupts the barriers while facilitating RNP rearrangements, a process which is necessary for efficient translation to proceed (Hartman et al., 2006; Bolinger et al., 2010; Ranji et al., 2011). DBPs can be targeted to RNA substrates through direct binding or interactions with protein components of targeted RNPs (reviewed in Jarmoskaite and Russell, 2011). In the case of RHA-dependent translation, RHA is targeted specifically to PCE-containing mRNAs through recognition of structural features within the PCEs, promoting translation (Hartman et al., 2006; Bolinger et al., 2010; Ranji et al., 2011). In the case of Lin28-dependent translation, RHA is likely targeted to LRE-containing mRNAs through interaction with Lin28, which recognizes and binds directly to the LREs (Jin et al., 2011, and reviewed in Huang, 2012).

### A working model

**Figure 3:**
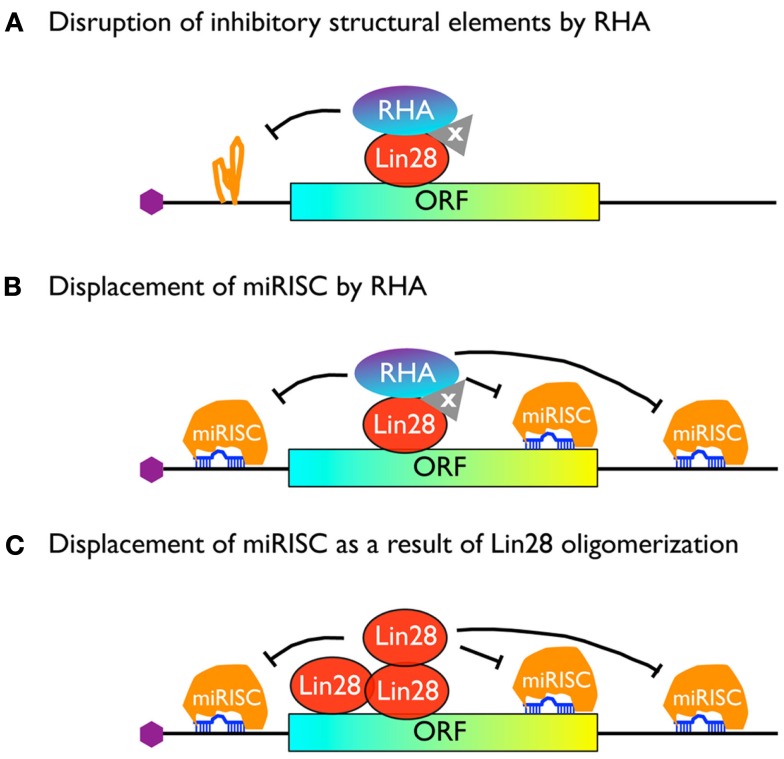
**Model for Lin28-mediated stimulation of translation**.

How might RHA promote translation after being recruited to Lin28 target mRNAs? One possibility is that RHA plays a role analogous to its interactions with PCE-containing mRNAs. In this regard, it is assumed that Lin28 target mRNAs contain yet uncharacterized structured RNA elements that reduce their efficiency of translation. Recruiting RHA to these mRNAs either directly by Lin28 or through interaction with a yet unknown factor(s; “protein x”) may overcome the inhibition by removing these structures, similar to the manner in which RHA disrupts the structures of PCEs (Figure 3A). An alternative (but not mutually exclusive) possibility is that Lin28 uses RHA as a motor to displace inhibitory protein-RNA complexes that bind to the mRNA, such as miRNA-induced silencing complexes, or miRISC, assuming that many or most Lin28 targets are also targets of miRNA inhibition (see below).

miRNAs are small non-coding RNAs that function to regulate gene expression mainly at the post-transcriptional level (reviewed in Fabian and Sonenberg, 2012). In mammals, hundreds of different miRNAs, which are predicted to control the expression of at least 50% of all genes, have been identified. Dysregulation of miRNA expression has been linked to human cancer and neural degenerative diseases (reviewed in Farazi et al., 2011; Chan and Kocerha, 2012). miRNAs regulate gene expression by binding to target mRNAs through base-paired interactions. Functional miRNA-binding sites have been found in various mRNA regions, including the 3′- and 5′-UTRs and the ORFs. miRNAs function in the form of miRISCs that contain Ago2 and GW182 as core effector components (reviewed in Fabian and Sonenberg, 2012). In mammalian cells, binding of miRISC to mRNAs leads in most cases to inhibition of translation and/or mRNA degradation. However, emerging evidence suggests that miRNA-mediated repression can be reversed under certain circumstances such as cellular stress or neuronal stimulation (reviewed in Filipowicz et al., 2008; Fabian and Sonenberg, 2012). For example, HuR binds to the AU-rich element (ARE) in the 3′-UTR of the cationic amino acid transporter mRNA (CAT-1) and impedes silencing of CAT-1 by miR-122 upon amino acid starvation (Bhattacharyya et al., 2006). Further studies using *in vitro* systems have suggested that this HuR-mediated derepression likely occurs via oligomerization along the RNA strand which leads to miRISC dissociation (Kundu et al., 2012).

Since one major function of DEAD-box RNA helicases is to unwind short RNA duplexes and cause displacement of RNA-protein complexes from a long RNA molecule, we propose that RHA recruited by Lin28 (either directly or through interaction with “protein x”) acts in an analogous way to displace miRISC from Lin28 target mRNAs (Figure 3B).

An alternative possibility is that Lin28 oligomerizes along its target mRNA following initial binding to an LRE and contributes to miRISC dissociation (Figure 3C). Several lines of evidence lend support to this theory. First, in our previous EMSA studies, purified recombinant Lin28 protein was mixed with individual LRE-containing RNA fragments and native gel electrophoresis was performed to examine protein-RNA complex formation. While Lin28-RNA complexes containing single Lin28 molecule could be readily detected at low Lin28 concentrations, supershifts were detected at higher Lin28 concentrations (Lei et al., 2011). Similar results were obtained with pre-let-7 RNA fragments known to interact with Lin28 both *in vitro* and *in vivo* (Desjardins et al., 2012). Second, NMR studies combined with RNA footprinting assays have suggested that Lin28-binding to a G-rich bulge within the pre-let-7 terminal loop partially unfolds the RNA, which enables multiple copies of Lin28 to bind to the RNA and protect the RNA from being cut by Dicer (Desjardins et al., 2012). Together, these observations suggest that Lin28 binds to multiple sites along the RNA (possibly in a cooperative manner) a property that may allow Lin28 to remove miRISC from the mRNA, much like the mechanism by which HuR displaces miRISC from ARE-containing mRNAs (Kundu et al., 2012).

In summary, we propose that the above three mechanisms may contribute to Lin28-mediated stimulation of translation, either singly or in combination, and dependent on cell type, developmental stage, and/or cell condition. Further studies will be required to thoroughly test this hypothesis.

## Conflict of Interest Statement

The authors declare that the research was conducted in the absence of any commercial or financial relationships that could be construed as a potential conflict of interest.
